# Origin and Development of Phloem and Xylem as Revealed in 3D Models of the Vascular Cylinder in Primary Roots of *Oryza sativa* L. cv. Hitomebore

**DOI:** 10.3390/plants15040607

**Published:** 2026-02-14

**Authors:** Yasushi Miki, Susumu Saito, Teruo Niki, Daniel K. Gladish

**Affiliations:** 1 Image Processing Section, MikiOn LLC, 593-1-102 Kunugidamachi, Hachioji 193-0942, Tokyo, Japan; yas@mikion.org (Y.M.); mdjmk210@ybb.ne.jp (S.S.); teruo-niki@hb.tp1.jp (T.N.); 2 Department of Biological Sciences, Miami University-Hamilton, 1601 University Blvd, Hamilton, OH 45011, USA

**Keywords:** initial cells, vascular differentiation, pericycle protoxylem origin, *Oryza sativa* L., virtual 3D model

## Abstract

Xylem and phloem are the defining features of vascular plants and are central to their evolutionary success. The origin and development of phloem and xylem within the vascular cylinder of rice primary roots (*Oryza sativa* L.) were investigated using serial sectioning and three-dimensional (3D) image processing techniques improved and developed by the authors. Protophloem mother cells and metaphloem sieve tube members were derived from vascular initials at about 20 μm from the apex of the vascular cylinder (AVC). Protophloem tissue and companion cells were generated through two successive divisions of protophloem mother cells 60–95 μm from the AVC of a 20 mm-long primary root. Metaxylem and late-metaxylem were initiated by the division of vascular initials at about 20 μm from the AVC of a 20 mm long root. Protoxylem was formed basipetally from secondary initials produced through periclinal division of pericycle cells at distances ranging 175–560 μm from the AVC. Protoxylem vessels could be clearly identified with a thickened cell wall preceding the thickening of metaxylem cell walls. Around 15 mm from the AVC of the 20 mm-long root, three kinds of xylem vessels (protoxylem, metaxylem and late metaxylem) were confirmed. The histogenesis of protophloem and protoxylem in rice primary roots was carefully analyzed and discussed in this study. In particular, the view has been confirmed regarding the pericycle origin of protoxylem in rice.

## 1. Introduction

The vascular system of plants, comprising phloem and xylem, does not simply define the anatomy of the autotrophic organisms that dominate terrestrial ecosystems, it serves as the critical conduit for the transport of essential substances that facilitate their complex life processes that make dominance possible. Therefore, the ontogenesis of the vascular cylinder has been of interest to many plant morphologists and developmental anatomists.

Over the last 160 years much work has been invested in evaluating and understanding the anatomical structure and patterns of histogenesis in root apical and primary meristem zones, beginning with von Hanstein’s “Histogen Theory,” which states that tissues are produced at organ apices by cell proliferation from three more-or-less permanent and distinct groups of cells called histogens, each responsible for the development of specific tissues [1]. Theories distinguishing “open” vs. “closed” root meristem organizations were described and debated [2,3,4]. Roots of cereal grain taxa have long been topics of research because their typically closed meristem organization simplifies the analysis of histogenesis [2,3,4,5,6,7]. Our general understanding of root apical meristem (RAM) structure and behavior was well reported by Williams [8] and reviewed by Jiang and Feldman [9] and Heimsch and Seago [10].

In their 1978 and 1979 investigation reports on the vascular cylinders of rice (*Oryza sativa* L.) crown roots, Kawata et al. [11,12] suggested a close correlation between the positions and “differentiation of each vessel and that of each sieve tube.” They demonstrated that protophloem sieve tube members, along with two companion cells, are formed via a pair of specific divisions of protophloem mother cells. Although the process of protophloem sieve tube differentiation was clearly described, the origin of the protophloem mother cells and the development of metaphloem was not reported.

The traditional understanding of xylem formation in the vascular cylinder is that the xylem is derived from specific promeristem cells located inside of the pericycle [8,13,14]. For example, key events in the histogenesis of metaxylem tissue were mapped by Feldman [15] in a commercial maize cultivar (*Zea mays* ‘Kelvedon 33’). He reported that the first four metaxylem files “are evident simultaneously” on average at about 65 µm from the “rootcap junction” (RCJ) and that metaxylem differentiation was correlated with “quiescent center” size.

Barley (*Hordeum vulgare*) and rice (*Oryza sativa*) are fairly closely related. They share a subclade in the Poaceae—the grass family—and they share many root-specific structural characteristics [14,16]. A century ago, Jackson [5] investigated the apical structure of barley roots and proposed that protoxylem originates from pericycle cells opposite developing metaxylem vessels (MX). According to her report, pericycle consists of radially elongated cells with uniformly thickened cell walls. Its continuity is interrupted opposite each xylem group, where protoxylem elements directly abut the endodermis [5]. Heimsch [6] also examined barley roots and interpreted micrographs to show that protoxylem elements are located adjacent to the metaxylem within the pericycle; i.e., at each xylem pole, a small protoxylem element differentiates in the cell layer commonly referred to as the pericycle. This protoxylem element is either in direct contact with the endodermis or separated from it by a similarly small cell, which is formed via a periclinal division that precedes protoxylem differentiation. In a report on phloem development, Warmbrodt [17] showed barley root anatomy data at approximately 21 cm along an elongated seedling root. Those data coincidentally showed the presence of protoxylem within the pericycle opposite where the metaxylem was positioned, and, unlike metaxylem cells, protoxylem elements lacked uniformly thickened cell walls. In those observations, protoxylem cells located within the pericycle were smaller than the adjacent pericycle cells.

Similar conclusions were drawn for rice. In their study on the vascular cylinders of rice crown roots, Kawata et al. [11] reported that radial divisions occur in pericycle cells adjacent to the metaxylem, leading to the differentiation of protoxylem elements and resulting in a localized interruption of pericycle continuity. Clark and Harris [18] and Scarpella et al. [19] also observed the anatomy of the roots of rice plants and identified small cells in the pericycle as protoxylem, but they did not explain their diagnostic criteria for protoxylem. In other words, the origin of protoxylem was not thoroughly addressed in their reports. A common feature across those observations was the presence of small cells at sites where early metaxylem elements were in contact with the pericycle; some of these small cells appeared to differentiate into protoxylem.

On the other hand, general descriptions of pericycle functions typically include initiation of lateral root primordia and (in species with secondary growth) production of periderm and contributions to initiation of vascular cambium [13,14]; they did not describe a role for it in the initiation of protoxylem. A recent, otherwise thorough, review of *Arabidopsis* pericycle functions that extensively reported details about pericycle cell division potential, the detectable differences between pericycle cells at protoxylem versus protophloem poles, and a proposed possible role for protoxylem in delivering hormonal signals to pericycle cells to induce lateral root initiation did not indicate a reverse role for the pericycle in the initiation of protoxylem vessel elements [20].

The specific details of the typical monocotyledon closed apical organization and the histogenesis of the xylem system of rice roots was thoroughly described in a comprehensive review by Morita and Nemoto [21]. In dicotyledonous plants such as *Arabidopsis* and morning glory (*Ipomoea*) that also have closed apical organization, protoxylem is formed during primary growth from the procambium, a tissue derived from the apical meristem. Typically, the primary xylem forms a central metaxylem aggregation with protoxylem positioned at xylem “points” radiating toward the periphery near the pericycle [22,23], respectively. While these species have closed apical organization in their root apices, we feel one must be cautious about extrapolating conclusions from studies of them to distantly related species such as rice. The spatial arrangement of xylem in monocotyledonous roots differs markedly from that in dicotyledons [8,13,14,21]. This raises the question of whether protoxylem formation in monocots, such as rice and barley, follows a similar developmental pathway as dicotyledons.

Protoxylem vessels typically initiate later but mature earlier than metaxylem and are characterized by annular or helical, lignified secondary cell wall reinforcements when mature [6,8,13,20]. The developmental relationship between protoxylem and metaxylem may serve as a basis for defining their respective identities.

Saito et al. [24] reported on the relationship between vascular initials subtending the VC’s plerome and late metaxylem (LMX) development in the vascular cylinder of *Zea mays*. While they clarified the origin of LMX elements, the developmental origin of early metaxylem and protoxylem was not addressed.

Those classic studies using traditional histological approaches were made challenging for their authors due to the limits imposed by the necessity of having to deduce three-dimensional (3D) anatomical features and events from two-dimensional (2D) data, such as typical tissue sections prepared for microscope study. Though recent advances in section preparation significantly improved that situation [25], the traditional analysis process remains arduous. To address this challenge, a method was recently developed to generate 3D virtual reconstructions of root anatomical features using micrographs of thin-sections from root apical meristem tissue and graphics manipulation software on personal computers [26,27]. This method was successfully implemented to enable a comprehensive, high-resolution 3D analysis of the structures of the promeristem and histogenesis of pericycle and late-metaxylem vessels in primary and nodal roots of maize (teosinte and sweetcorn), barley, and rice [16]—the first of a planned series of studies to evaluate histogenesis of all the root procambial tissues of these important agronomic species using 3D analysis.

In barley, protoxylem elements have not been shown to form outside the pericycle layer, and cell division within this layer has been documented [6]. In contrast, the origin and developmental pattern of protoxylem in rice roots appears to be more complex than in barley [21]. We therefore thought it necessary to undertake an investigation of the origin of protoxylem formation in roots of graminaceous plants by our new methods.

High-resolution section preparation techniques [25] and three-dimensional (3D) image reconstruction from serial sections [26,27] are effective for elucidating the spatial configuration of cellular features. These methods will potentially enable more refined analyses of cell files and the developmental origin of xylem—particularly protoxylem—within the vascular tissue of the root apex. Our objective for the present study was to use these new methods to better understand the origins of protophloem and protoxylem in rice roots to confirm or reject prior (“classical”) interpretations based on conventional histological microscopic analysis.

## 2. Results

### 2.1. Plerome and “Initial Cell” Layer at the Vascular Cylinder Apex

**Figure 1:**
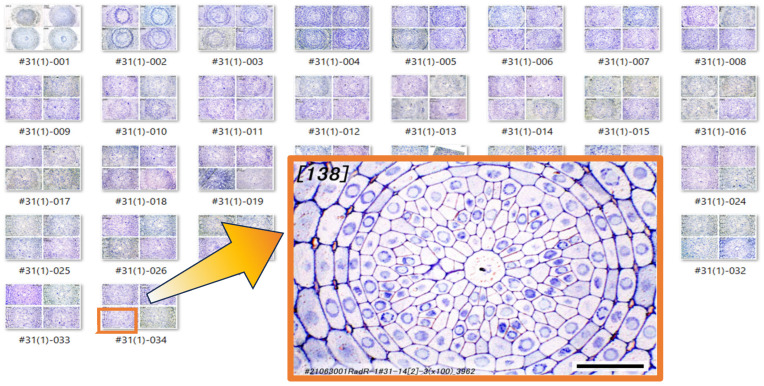
A series of original photo images of transverse sections through the vascular cylinder (VC). An enlarged image is included to highlight anatomical detail at a position 140 µm basipetal from the apex of the vascular cylinder (AVC). Scale bar = 30 µm.

**Figure 2:**
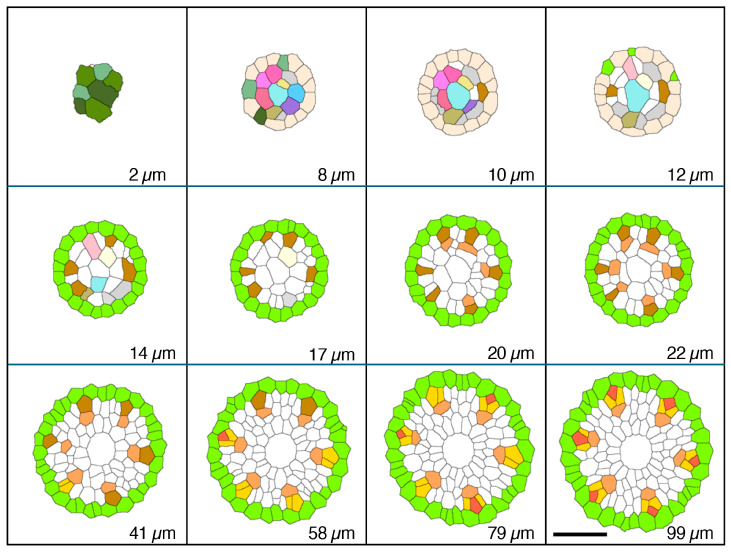
Micrographic images produced from a set of 1 µm-thick transverse serial sections modified to reveal cell wall patterns and colorized to feature specific procambial cell types (phloem focus), presented in order of increasing distance from the apex of the vascular cylinder (AVC). Plerome cells (2 µm, shades of green); initials layer (8–12 µm, various colors); onset of pericycle (GreenYellow) and phloem mother cells (DarkGoldenrod) file development (14 µm); beginning of metaphloem (20 µm, SandyBrown) cell files; first initiation of protophloem group cells (41 µm, Gold); first initiation of a protophloem sieve tube member (58 µm, Tomato) with companion cells (Gold); and all protophloem groups complete (99 µm). Scale bar = 50 µm.

**Figure 3:**
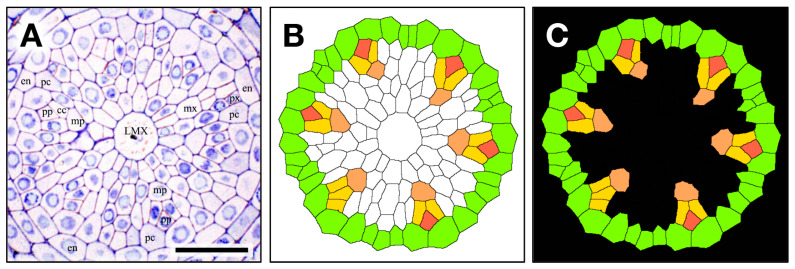
The contour extraction (**A**); cc, companion cell; en, endodermis; pc, pericycle; LMX, late metaxylem; mp, metaphloem; mx, metaxylem; pp, protophloem; px, protoxylem), coloring step (**B**) pericycle (GreenYellow), metaphloem (SandyBrown), protophloem (Tomato) companion cell (Gold), and introduced transparency procedure (**C**, black) for a 1 μm thick section used to construct a 3D virtual object. Scale bar = 30 µm.

**Figure 4:**
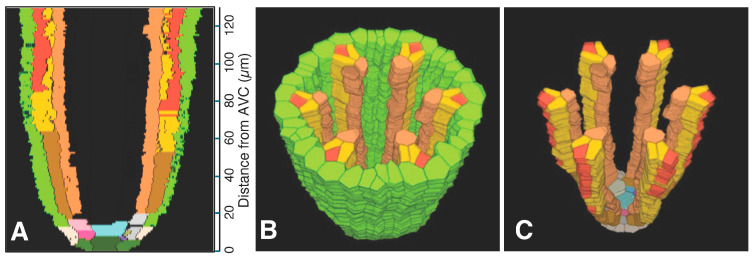
Phloem system. A three-dimensional object was reconstructed from serial sections of a rice vascular cylinder (VC). View angle from above: 60°. Pericycle (GreenYellow), protophloem mother cells (DarkGoldenrod), protophloem sieve tube (Tomato), sieve tube companion cells (Gold), and metaphloem sieve tubes (SandyBrown). Selected initials and plerome cells are shown in various distinct colors at the apex. The apex of the vascular cylinder (AVC) = 0 on the scale. (**A**) Virtual sectional view of the 3D reconstruction. (**B**) Bird’s-eye view of the 3D reconstruction. (**C**) Bird’s-eye view of the 3D reconstruction with the pericycle removed.

Figure 1 shows a chart of the original vascular cylinder serial 1 µm-thick sections to be modified to make a 3D reconstruction of the vascular tissue in a rice primary root tip. An enlarged image is included to highlight anatomical detail enhanced by enzyme treatment and staining at a position 140 µm basipetal from the apical vascular cylinder (AVC). Cell walls in these sections (Figure 2 and Figure 3B) were extracted using the ‘Find Maxima’ function in ImageJ 2.3.0, and pericycle cells, protophloem, companion cells, and meta-phloem were colorized (Figure 2 and Figure 3). The steps involved in preparing sections for building the 3D reconstruction are specifically shown in Figure 3. Each section was rendered transparent (Figure 3C) to allow the construction of a virtual 3D structure (Figure 4; Supplementary Figures S1 and S2 and Video S1: the phloem system). Our 3D reconstruction analysis confirmed that the tip of the rice root’s vascular cylinder apex was occupied by its plerome histogen, which was surrounded on the plerome margin by pericycle initials and was subtended basipetally by a layer of vascular tissue initials. Some of those had divided transversely to form files of differentiating xylem vessel elements, differentiating metaphloem sieve tube members, or secondary initials that ultimately produced protophloem mother cells and vascular parenchyma tissue (Figure 2 and Figure 4C).

### 2.2. Origin and Differentiation of Phloem

In the typical rice primary root tip shown in Figure 2, Figure 3 and Figure 4, the phloem conducting system development began when specific cells in the initials layer adjacent to the developing pericycle divided transversely. The basipetal daughters of those initials then usually divided periclinally to the pericycle to produce a protophloem mother cell to the outside (adjacent to the pericycle) and a metaphloem sieve tube initial to the inside, 10–22 µm from the AVC (Figure 2, “DarkGoldenrod” and “SandyBrown,” respectively). Six protophloem mother cells and metaphloem files were consistently produced (Figure 2, Figure 3 and Figure 4). The sieve tube initial typically divided in various longitudinal planes. A resulting daughter cell in contact with the protophloem mother cell became an incipient metaphloem sieve tube member (“SandyBrown”). Protophloem mother cell files proliferated by transverse division from their apical ends as root growth proceeded. The mother cells began protophloem differentiation at varying distances between ca. 40 and 60 µm from the AVC (Figure 2) by initiating, first one and then another, asymmetrical longitudinal cell divisions that resulted in a group of three cells, a protophloem sieve tube member adjacent to the pericycle (“Tomato”), and two companion cells (“Gold”) intervening between the developing proto- and metaphloem sieve tube members. This pattern was established in all six phloem strands by 99 µm from the AVC (Figure 2 and Figure 3). Contrary to Kawata et al. [11], it is worth noting that this pattern was not always generated sequentially in time such that the protophloem sieve tube file was always continuous at the acropetal end during its production (Figure 4C, Supplementary Video S1: the phloem system). However, the appearance and location in transverse sectional view and in 3D reconstruction of each protophloem group (a sieve tube member and two companion cells), as shown in Figure 3 and Figure 4, was dependably consistent and continuous at maturity.

### 2.3. Origin and Differentiation of Xylem

**Figure 5:**
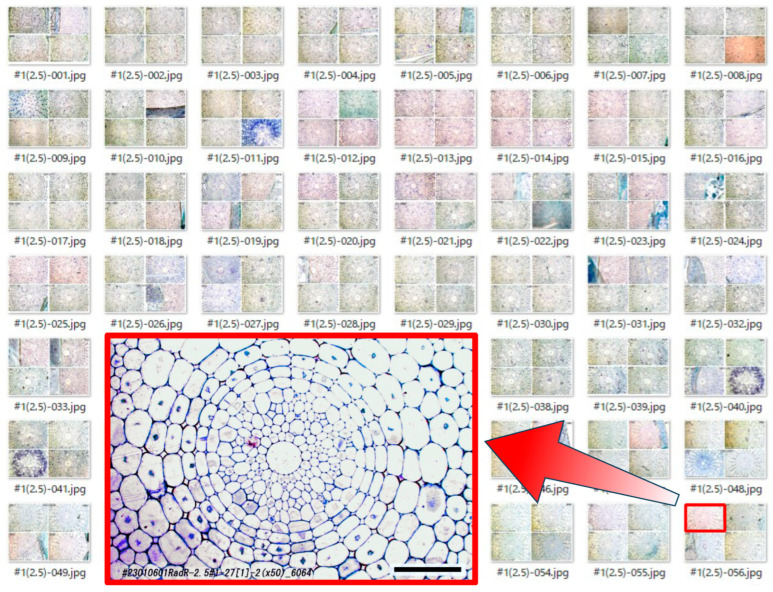
A series of original photo images of 2.5 µm-thick transverse sections through the vascular cylinder (VC). An enlarged image is included to highlight anatomical detail at a position 675 µm basipetal from the apex of the vascular cylinder (AVC). Scale bar = 50 µm.

**Figure 6:**
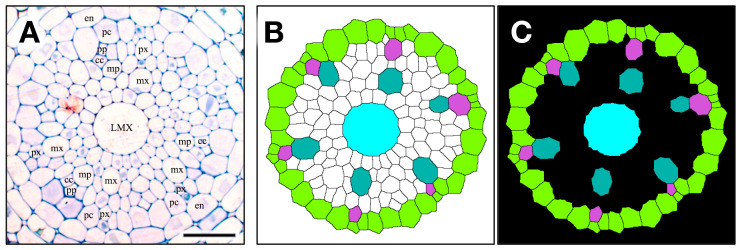
The contour extraction (**A**), (cc, companion cell; en, endodermis; pc, pericycle; LMX, late metaxylem; mp, metaphloem; mx, metaxylem; pp, protophloem; px, protoxylem); coloring step (**B**), pericycle (YellowGreen), metaxylem vessels (LightSeaGreen), late metaxylem vessel (LMX, Cyan), protoxylem vessel (MediumOrchid); introduced transparency procedure (**C**, black) for a 2.5 μm thick section used to construct a 3D virtual object. Scale bar = 25 µm.

**Figure 7:**
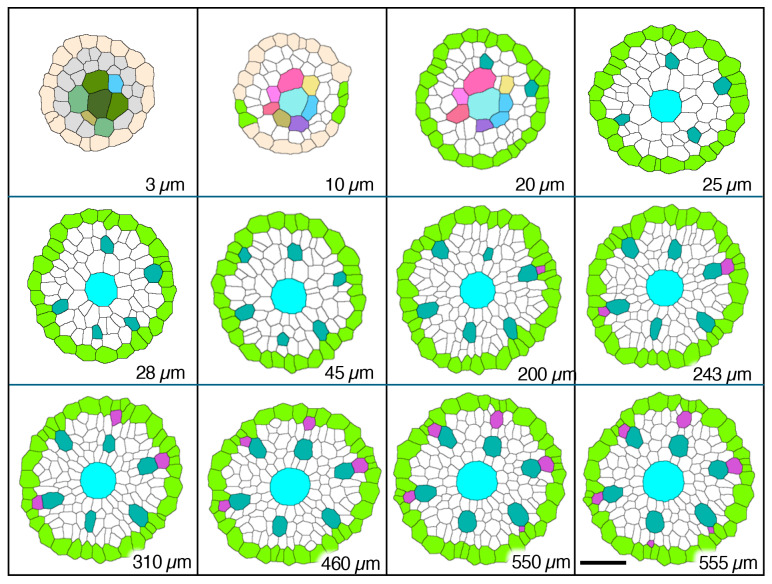
Micrographic images produced from a set of 2.5 µm-thick transverse serial sections modified to reveal cell wall patterns and colorized to feature specific procambial cell types (xylem focus), presented in order of increasing distance from the apex of the vascular cylinder (AVC). Plerome cells (3 µm, different shades of green) and plerome margin initials (AntiqueWhite); initials layer (10 µm, various colors) and first pericycle cells (YellowGreen); initiation of first metaxylem vessel elements (20 µm, LightSeaGreen); additional metaxylem vessel elements present (25 µm, LightSeaGreen) and late metaxylem vessel element (LMX, Cyan); first pericycle cell divisions to form protoxylem vessel elements (200 µm, MediumOrchid); all protoxylem points established (555 µm, MediumOrchid). Scale bar = 25 µm.

Serial sections 2.5 µm thick were used to analyze meta- and protoxylem vessel development, which required evaluation of VC tissues farther from the AVC than for protophloem (Figure 5), though the procedure for producing a 3D reconstruction for that purpose was the same as for phloem (Figure 6). Late-maturing metaxylem vessel (LMX) differentiation in rice primary roots began immediately with a daughter cell from the initial cell centrally located in the initials layer 10 µm from the AVC (“Cyan”; Figure 7). By 20 µm from the AVC, cell divisions by initial cells in the initial cell layer or subsequent divisions by their daughter cells gave rise to secondary initials that produced files of metaxylem vessel elements (“LightSeaGreen”; Figure 7). Typically, there were six such files in primary roots, the majority of which were adjacent to the pericycle.

**Figure 8:**
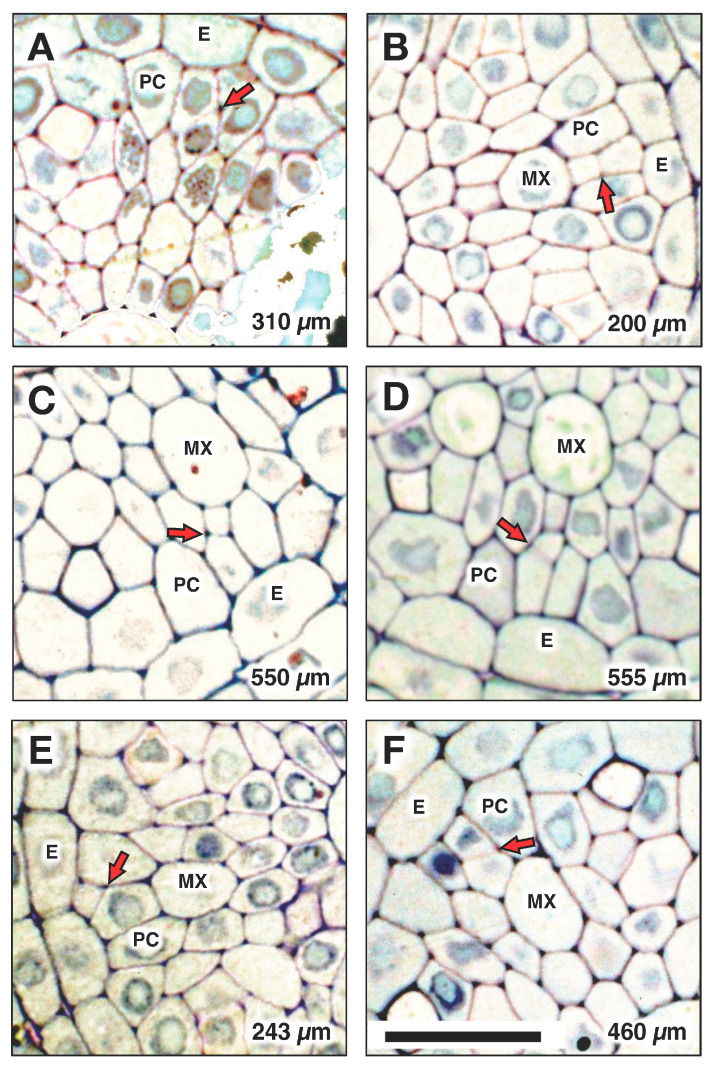
Locations of protoxylem initiation from a pericycle cell division in one root are indicated at the following distances from the apex of the vascular cylinder (AVC): (**A**) 310 µm, (**B**) 200 µm, (**C**) 550 µm, (**D**) 555 µm, (**E**) 243 µm (spanned the PC), (**F**) 460 µm. Arrows indicate the cell division that initiated protoxylem differentiation. E: endodermis; PC: pericycle; MX: metaxylem. Scale bar = 20 µm.

**Figure 9:**
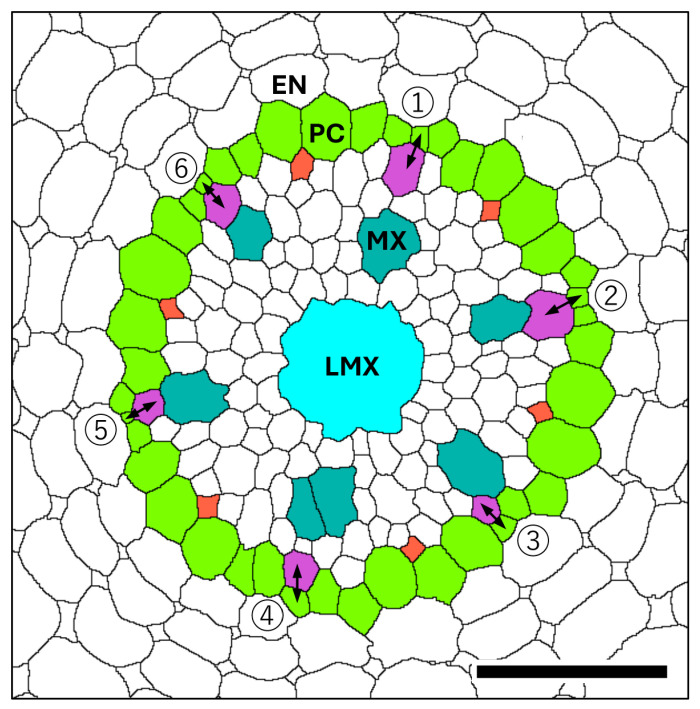
Diagram of the vascular cylinder (VC) at a position 1.3 mm basipetal from the apex of the vascular cylinder (AVC). Endodermis (EN), pericycle (PC, GreenYellow), late metaxylem vessel (LMX, Cyan), metaxylem vessel (MX, LightSeaGreen), protoxylem (MediumOrchid), protophloem (Tomato). Protoxylem initiation from pericycle cell divisions (double-headed arrows) for specific protoxylem elements in this root (see Figure 8) were observed at the following distances from the AVC: ① 310 µm, ② 200 µm, ③ 550 µm, ④ 555 µm, ⑤ 243 µm, and ⑥ 460 µm. Scale bar = 50 µm.

**Figure 10:**
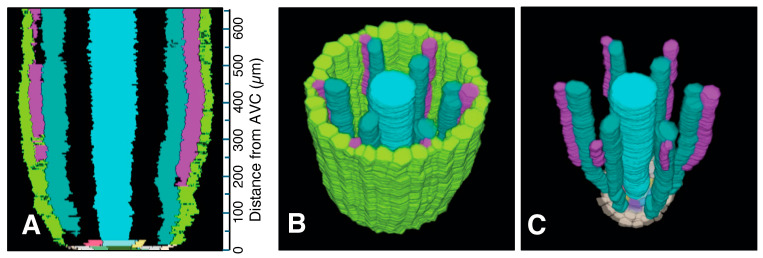
Xylem system. A three-dimensional object reconstructed from serial sections of the vascular cylinder (VC). View angle from above: 60°. Pericycle (GreenYellow), protoxylem vessels (MediumOrchid), metaxylem vessels (LightSeaGreen), and late metaxylem vessel (Cyan). Selected initials and plerome cells are shown in other distinct colors. AVC = apex of the vascular cylinder. The AVC = 0 on the scale. (**A**) Virtual sectional view of the 3D reconstruction. (**B**) Bird’s-eye view of the 3D reconstruction. (**C**) Bird’s-eye view of the 3D reconstruction with the pericycle removed.

**Figure 11:**
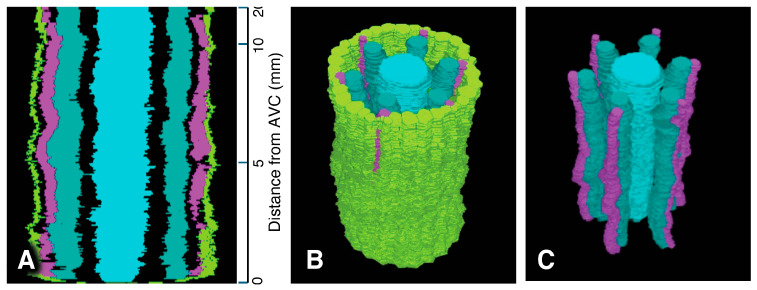
Xylem system. A three-dimensional object reconstructed from serial sections of the vascular cylinder (VC), spanning from 0 to 20 mm basipetal from the apex of the vascular cylinder (AVC). View angle from above: 46°. Pericycle (GreenYellow), protoxylem vessels (MediumOrchid), metaxylem vessels (LightSeaGreen), and late metaxylem vessel (Cyan). AVC = 0 on the scale. (**A**) Sectional view of the 3D reconstruction. (**B**) Bird’s-eye view of the 3D reconstruction. (**C**) Bird’s-eye view of the 3D reconstruction with the pericycle removed.

**Figure 12:**
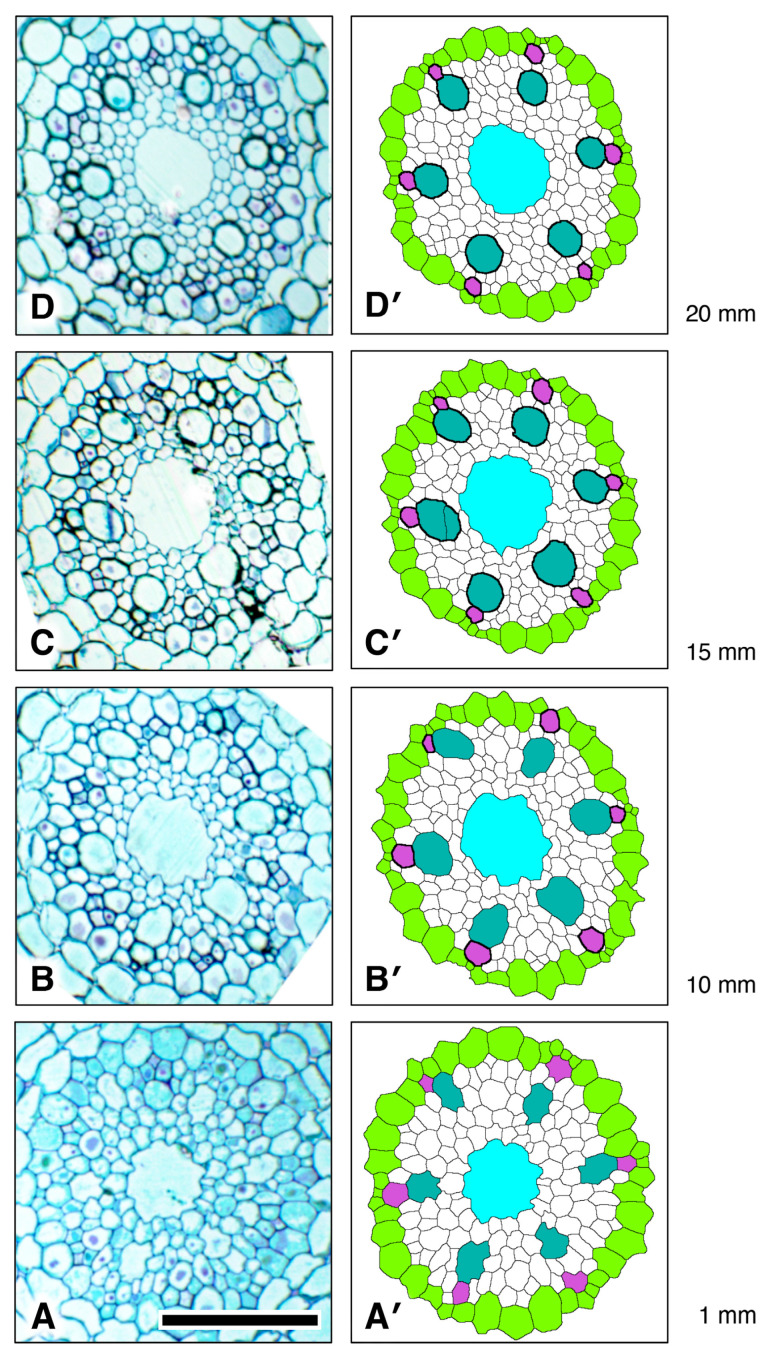
Xylem vessel cell wall thickening trends during development in the procambium of a rice primary root showing that protoxylem cell wall thickening occurs sooner than in metaxylem and much sooner than late metaxylem. (**A**,**A’**) Original micrograph and processed, colorized micrograph, respectively, taken at 1 mm from the apex of the vascular cylinder (AVC). (**B**,**B’**) Original section micrograph and processed, colorized micrograph, respectively, taken at 10 mm from the AVC. (**C**,**C’**) Original section micrograph and processed, colorized micrograph, respectively, taken at 15 mm from the AVC. (**D**,**D’**) Original section micrograph and processed, colorized micrograph, respectively, taken at 20 mm from the AVC. Pericycle (GreenYellow), protoxylem vessels (MediumOrchid), metaxylem vessels (LightSeaGreen), and late metaxylem vessel (Cyan). Locations of thickened vessel walls are indicated in the colorized images by thicker, darker cell walls. Scale bar = 50 µm.

Associated with each metaxylem file and beginning at various distances from the AVC, a longitudinal cell division of a pericycle cell (Figure 8) gave rise to a secondary initial cell that would proliferate longitudinally during root growth to produce a file of protoxylem vessel elements (“MediumOrchid”; Figure 9 and Figure 10). Occasionally the protoxylem cells would span the pericycle resulting in a zone of interrupted pericycle (Figure 10A and Figure 11B). Sometimes parenchyma cells were found located between meta- and protoxylem cell files (Figure 9, Figure 10 and Figure 12).

We determined the longitudinal distribution of secondary wall initiation by protoxylem (“Orchid”) and metaxylem vessels in the VC of a rice primary root grown to 20 mm length under laboratory conditions (Figure 12). There was no visible increase in wall thickness in either cell type less than 1 mm from the AVC. By 10 mm from the AVC protoxylem vessels had thickened walls and were likely fully functional. Cell walls of metaxylem vessels (“LightSeaGreen”) were thickened at 15 mm from the AVC, but vessel elements in that location had cytoplasmic contents, therefore they were not yet functional. Although the secondary walls of metaxylem of all six metaxylem vessels were well thickened at 20 mm from the AVC, several vessels still retained cytoplasm (Figure 12). In the region of the VC we studied, the central LMX vessel (“Cyan”) showed no sign of secondary wall formation and retained cytoplasmic contents (Figure 12).

Colorized serial sections enabled the reconstruction of a three-dimensional image of the VC (Figure 10), allowing clearer visualization of xylem origin and development. A virtual longitudinal view of the VC revealed cellular features within the pericycle, along with protoxylem and metaxylem. Colorized serial sections enabled the reconstruction of a three-dimensional image of the VC (Figure 10), allowing clearer visualization of xylem origin and development. A virtual longitudinal view of the VC revealed cellular features within the pericycle, along with protoxylem, metaxylem and late metaxylem elements (Figure 10A). Figure 10B,C present 3D reconstructions of the VC with xylem, shown with and without the pericycle, respectively (also see Supplementary Figures S3 and S4 and Video S2: xylem system).

A total of 8000 2.5 µm-thick transverse serial sections from a 20 mm-long primary root were carefully collected sequentially to examine the root vascular anatomy and cell division patterns of the pericycle. Figure 9 summarizes the spatial distribution of pericycle cell divisions opposite the metaxylem relative to the AVC in that root. A 3D reconstruction of this specimen was generated (Figure 11; Supplementary Figures S5 and S6). This 3D construct shows the locations of protoxylem vessels resulting from cell division activity of the pericycle.

## 3. Discussion

### 3.1. Verifying and Enhancing Classical Interpretations of Vascular Development in Oryza Primary Roots

As part of an ongoing effort to take advantage of newly developed histological preparation techniques for light microscopes and virtual 3D reconstructions to analyze developmental patterns at a high resolution, we are working to verify classical theories and models of histogenesis in developing roots. The new approach to histochemical preparation [25] clarifies cell boundaries and internal tissue patterns in cytoplasmically dense meristematic tissues typically found in the apical zones of growing roots. The use of digital 3D virtual reconstruction [26,27] affords a greater appreciation of the spatial relationships of specialized tissue structures within defined tissue zones, such as xylem and phloem sectors, and their origins within the procambium. Furthermore, it allowed us to observe some details that might have been overlooked by traditional methods.

By conventional microscopy, the traditional method [24], enhanced by new histological methods [25], revealed that all metaxylem cell files (early and late) could ultimately be traced to a layer of initial cells basipetal to the plerome in primary roots of *Zea mays*. The present report shows a similar result for *Oryza sativa*, but the details can more easily be seen and appreciated by readers; all relative distances from the AVC along cell files can be seen at once, as can the “trajectories” (morphology) of all cell files included in each 3D reconstruction. This, of course, means that the discretion of the researcher as to what to include in a 3D reconstruction is important.

On the other hand, all the original data are conserved and can be accessed to provide other observational options later, such as presenting protoxylem point or protophloem group data separately, as shown here (Figure 4 vs. Figure 10) or the VC with or without the pericycle (Figure 4, Figure 10 and Figure 11).

### 3.2. Origin and Development of Phloem Sieve Tubes and Companion Cells

As mentioned above, the origin of metaphloem and protophloem mother cell files had not previously been carefully described in the roots of these taxa. By careful serial sectioning, slide preparation, digital photomicroscopy, and management of those micrographs in order to “reconstruct” the original tissues as 3D virtual in silico objects, our analysis showed how protophloem—via the action of protophloem mother cell division—and metaphloem sieve tube members are derived from the initials at the AVC in the primary roots of *Oryza sativa* ‘Hitomebore’.

### 3.3. Origin and Development of Xylem Vessels

We believe that accurate, detailed mapping of tissue structures, such as vascular fluid conducting cell files (e.g., sieve tubes and xylem vessels), is useful to molecular genetics researchers because it allows precise localization of key developmental switch-points. Good examples are related to the “transcription switches” that trigger and coordinate the differentiation of root apical meristem cells into meta- and protoxylem vessel elements that are in the VASCULAR-RELATED NAC-DOMAIN (VND) gene family. VND6 and VND7 are implicated in the initiation of cell differentiation of meta- and protoxylem vessel elements, respectively. The production of these transcription factors is dependent on the presence of auxin, cytokinin, and brassinosteroid hormones downstream from their sources [28], and doubtless depend on the concentrations and the relative ratios of those among them. Much has been learned in the 15 yr since the publication of those results. It is worth remembering that the studies were conducted using the dicotyledon taxa, *Zinnia* and *Arabidopsis* [29], as this demonstrates our initial claim of the value of detailed histological mapping, since *Arabidopsis* root histogenesis and anatomy are well understood and mapped [22].

The proper differentiation of protoxylem vessels is essential to a plant’s successful growth [30,31]. As the roots elongate, protoxylem plays an important role in carrying water and regulatory signals to developing meristematic cells while stretching as a consequence of axial growth, therefore it needs to be both elastic and functional [13,14,30,31]. It has also been reported that under stresses, such as dryness and salt exposure, the development of protoxylem changes [30,32].

Auxin is present at relatively high concentration at the apex of the root body, and it controls cell division and differentiation in combination with other regulatory hormones, such as cytokinin. In particular, in the process of differentiating xylem progenitor cells into mature tracheary elements, the concentration gradient of auxin plays an important role [33]. In the root VC, auxin is transported acropetally through phloem sieve tubes (probably mainly as a conjugate) and by polar, cell-to-cell transport through living immature metaxylem vessel elements, surrounding parenchyma cells, and pericycle cells [33].

Although they become completely differentiated and functional before the metaxylem cells do, the protoxylem elements first become distinguishable farther from the AVC than the metaxylem. The completion of full differentiation of metaxylem vessel elements is often located at the basal end of the root extension zone, just before where root hairs begin to develop [8,13].

In the present study, we demonstrated that protoxylem in rice does not originate from within the VC promeristem, but rather from cell divisions within the pericycle ranging from 175 to 560 μm distributed basipetally from the AVC (Figure 8 and Figure 10A). A notable feature of this process is that the division takes place in regions of the pericycle adjacent to developing metaxylem, suggesting that metaxylem proximity possibly influences pericycle cell divisions that lead to protoxylem initiation. On the other hand, since root phloem is reported to be a major source of auxin from the shoot system, and gradients of auxin concentration are implicated in the differentiation of xylem tracheary elements [33], we speculate that the location of protoxylem initiation midway between functional protophloem sieve tubes may be a consequence of local variations in auxin concentration by virtue of phloem-contributed auxin.

Some of the daughter cells resulting from pericycle cell division subsequently undergo secondary cell wall thickenings to form protoxylem elements, which can typically be clearly seen 10 mm from the AVC (Figure 12B). Such wall thickening occurs prior to the thickening of metaxylem cell walls, which is clearly underway 15 mm from the AVC (Figure 12C) and which indicates that axial root elongation is no longer happening, though the presence of cytoplasm in the metaxylem elements indicates that they are not yet fully mature and functional. Both protoxylem and metaxylem elements exhibit wall thickening at 20 mm from the AVC, whereas late metaxylem vessel walls remain unthickened. This developmental stage indicates that the seedling root has not yet reached full maturation.

**Figure 13:**
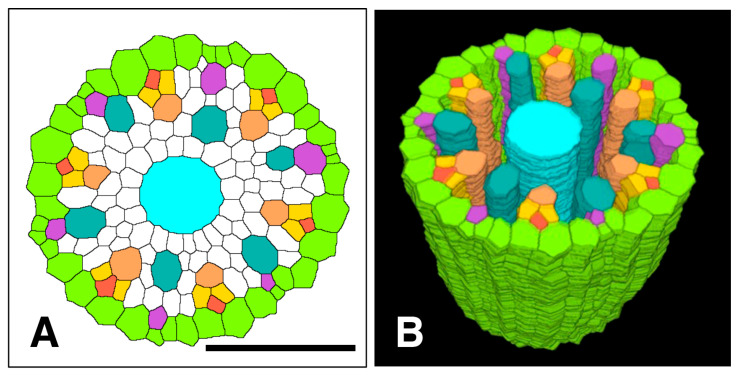
Spatial distribution of all phloem and xylem cells with respect to the pericycle in the apex of the procambium of a rice primary root. (**A**) Processed and colorized transverse section taken 650 µm from the apex of the vascular cylinder, (**B**) 3D reconstruction of the apical pericycle, phloem and xylem tissues. Pericycle (GreenYellow), protophloem mother cells (DarkGoldenrod), protophloem sieve tubes (Tomato), sieve tube companion cells (Gold), metaphloem sieve tubes (SandyBrown), protoxylem vessels (MediumOrchid), metaxylem vessels (LightSeaGreen), and late metaxylem vessel (Cyan). Scale bar = 50 µm.

**Figure 14:**
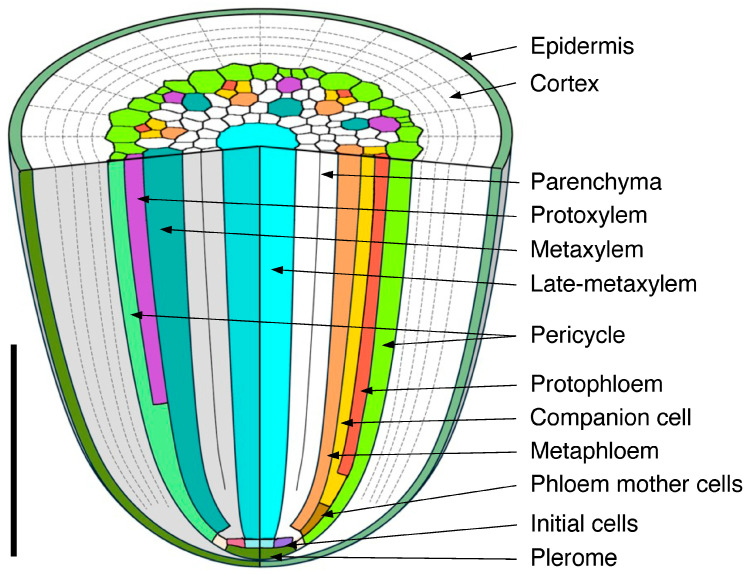
Summary schematic diagram of vascular tissue development in a primary root of rice. Scale bar = 500 µm.

Although the preparation of quality conventional microscope slides for LM requires experience and skill, and is somewhat laborious, the use of advanced histological methods and image processing makes the conversion of serial digital images into 3D virtual objects that can be manipulated in various ways to reveal meaningful information about the original tissues possible (Figure 13). A summary schematic diagram of our conclusions about the origins and histogenesis of the conducting tissues of the rice primary root is given in Figure 14. We intend further analyses involving auxin inhibitor treatments to help elucidate the role of hormonal regulation underlying these anatomical transitions. We also intend to use this new approach to explore histogenesis in a dicotyledonous taxon with closed apical organization, such as *Ipomoea*.

## 4. Materials and Methods

### 4.1. Plant Materials

Rice (*Oryza sativa* L. cv. ‘Hitomebore’) was used. The Hitomebore cultivar was used because it is prized by Japanese consumers for having many desirable culinary qualities. Cultivation methods were modified after Gladish and Niki [34]. Seeds were surface sterilized in 10% household bleach, sown in moistened, autoclaved vermiculite, and then incubated in constant 25 °C in a continuously dark growth chamber for 3–4 d.

### 4.2. Preparation for Light Microscopy (LM)

The procedures used for LM were modified from Niki et al. [35]. Root tip segments of rice primary roots were taken from the selected roots (2.2 ± 0.2 cm in length), immediately immersed in 4% (*w*/*v*) paraformaldehyde in 0.1 M phosphate buffer (pH 7.2) and gently shaken overnight at room temperature. Following fixation, the specimens were rinsed in the buffer, dehydrated in an ascending ethanol series (50, 75, 90, 100, 100%) for 30 min each, and embedded in Technovit 7100 resin (Heraeus Kulzer GmbH, Wehrheim, Germany) in a graded ethanol:resin series (50:50, 25:75, 0:100%) for 1–2 hr each; both processes were gently shaken while performed. Serial sections were made transversely with a glass knife at a thickness of 1 or 2.5 μm on a Reichert-Nissei UCT ultramicrotome (Leica Ltd., Tokyo, Japan). The sections were placed in serial order on APS coated Micro-slide glass slides (Matsunami Glass Ind., Ltd., Tokyo, Japan) and dried.

The sections were then treated with RNase A solution (Sigma Chemical Co., St. Louis, MO, USA), 60 µL/300 µL distilled water each, to increase contrast and definition of cell wall patterns in the cytoplasmically dense meristematic tissue [25]. After enzyme treatment, the sections were stained with 0.1% (*w*/*v*) toluidine blue O (TB) (Electron Microscopy Sciences, Hatfield, PA, USA) at 45 °C for 10 min and rinsed with distilled water. The sections were observed uncovered with a Leica DMLB light microscope (Leica Microsystems GmbH, Wetzlar, Germany) equipped with TU Plan Fluor lenses (20× ∞/0 or 50× ∞/0, Nikon Corp., Tokyo, Japan) and photographed with a Canon EOS 5D Mark II digital camera (Canon Inc., Tokyo, Japan), according to Niki et al. [25].

### 4.3. Image Processing

Digital images of serial sections were acquired with a resolution of 1404 × 936 pixels (downsized from the original camera resolution of 5616 × 3744 pixels), which corresponded to 270 × 180 µm when the 50× objective was used. GIMP 2.10 (S. Kimball, P. Mattis and the GIMP Development Team) and ImageJ 2.3.0 (U. S. National Institutes of Health) running on a MacBook laptop computer (Apple Inc., Cupertino, CA, USA) were employed for image processing. These image manipulation applications are open-source and available to the public for free. The first step was the contour extraction from photomicrographs using the “Find Maxima” tool in ImageJ. The second step was to colorize specific cells (protophloem, metaphloem, protoxylem, metaxylem, late-metaxylem, pericycle cells, and their initial cells, etc.) by using the “Bucket Fill” tool of GIMP in order to distinguish them from other cells. The third step was to align the images with each other. These steps were essentially identical to those described previously [26,27]. In this study, however, an automatic alignment tool—the “Registration” plugin provided by ImageJ—was effectively utilized [36]. The 3D virtual objects were constructed from the stacked and aligned images using the “3D Viewer” plugin of ImageJ. As in our previous work [16], colors used for images were all from the 140 standard HTML color series (https://www.w3schools.com/tags/ref_colornames.asp, accessed on 15 December 2025).

## 5. Conclusions

We confirmed that, in the primary roots of the Hitomebore cultivar of *Oryza sativa*, phloem initials immediately basipetal to the plerome adjacent to the pericycle produce a file of cells that soon divide periclinally to form incipient metaphloem sieve tube elements to the inside and protophloem mother cells to the outside, adjacent to the pericycle. The mother cells each divide again axially twice in sequence at oblique angles that usually result in a protophloem sieve cell against the junction of two pericycle cells to the outside and two companion cells that separate it from the metaphloem sieve tube. Contrary to previous reports on rice root phloem histogenesis, this distinctive pattern does not always occur continuously in the acropetal direction, such that gaps in continuity of the nascent protophloem sieve tube are apparent. These are eliminated later as the protophloem sieve tube complex matures.

We confirmed that protoxylem vessel elements arise from axial anticlinal and periclinal cell divisions of pericycle cells. Contrary to previous reports, the resulting vessel does not always interrupt the continuity of the pericycle layer. Metaxylem files are produced continuously from initials located in the initials layer immediately basipetal to the plerome.

Consistent with the vast majority of descriptions of primary root xylem development, *O. sativa* metaxylem initiates before protoxylem, but the metaxylem does not mature until after axial growth ceases; whereas, protoxylem matures and becomes functional in the root’s elongation zone.

## Data Availability

The raw data supporting the conclusions of this article will be made available by the authors on request. The data are not publicly available due to cybersecurity concerns.

## Supplementary Materials

The following supporting information can be downloaded at: https://www.mdpi.com/article/10.3390/plants15040607/s1, Figure S1: Set of 1 µm-thick serial sections colorized after contour extraction (Figure 3B) used to construct a 3D representation of the phloem system of a rice root. Figure S2: Set of 1 µm-thick colorized serial sections (Supplementary Figure S1) after transparency was introduced to construct a 3D representation of the phloem system of a rice root. Figure S3: Set of 2.5 µm-thick serial sections colorized after contour extraction (Figure 6B) used to construct a 3D representation of the xylem system of a rice root. Figure S4: Set of 2.5 µm-thick colorized serial sections (Supplementary Figure S3) after transparency was introduced to construct a 3D representation of the xylem system of a rice root. Figure S5: Set of 2.5 µm-thick serial sections colorized after contour extraction (Figure 11B) used to construct a 3D representation of the xylem system of a rice root. Figure S6: Set of 2.5 µm-thick colorized serial sections (Supplementary Figure S5) after transparency was introduced to construct a 3D representation of the xylem system of a rice root. Video S1: Phloem system of rice primary root apex. Video S2: Xylem system of rice primary root apex.
