# Supplementary material for: Origin and Development of Phloem and Xylem as Revealed in 3D Models of the Vascular Cylinder in Primary Roots of Oryza sativa L. cv. Hitomebore
Source: Plants (Basel). 2026 Feb 14;15(4):607. doi: 10.3390/plants15040607 (PMC12944624; doi:10.3390/plants15040607)
Supplement: Supplementary file 1 [file plants-15-00607-s001.zip › SupplFigureS1.pdf]

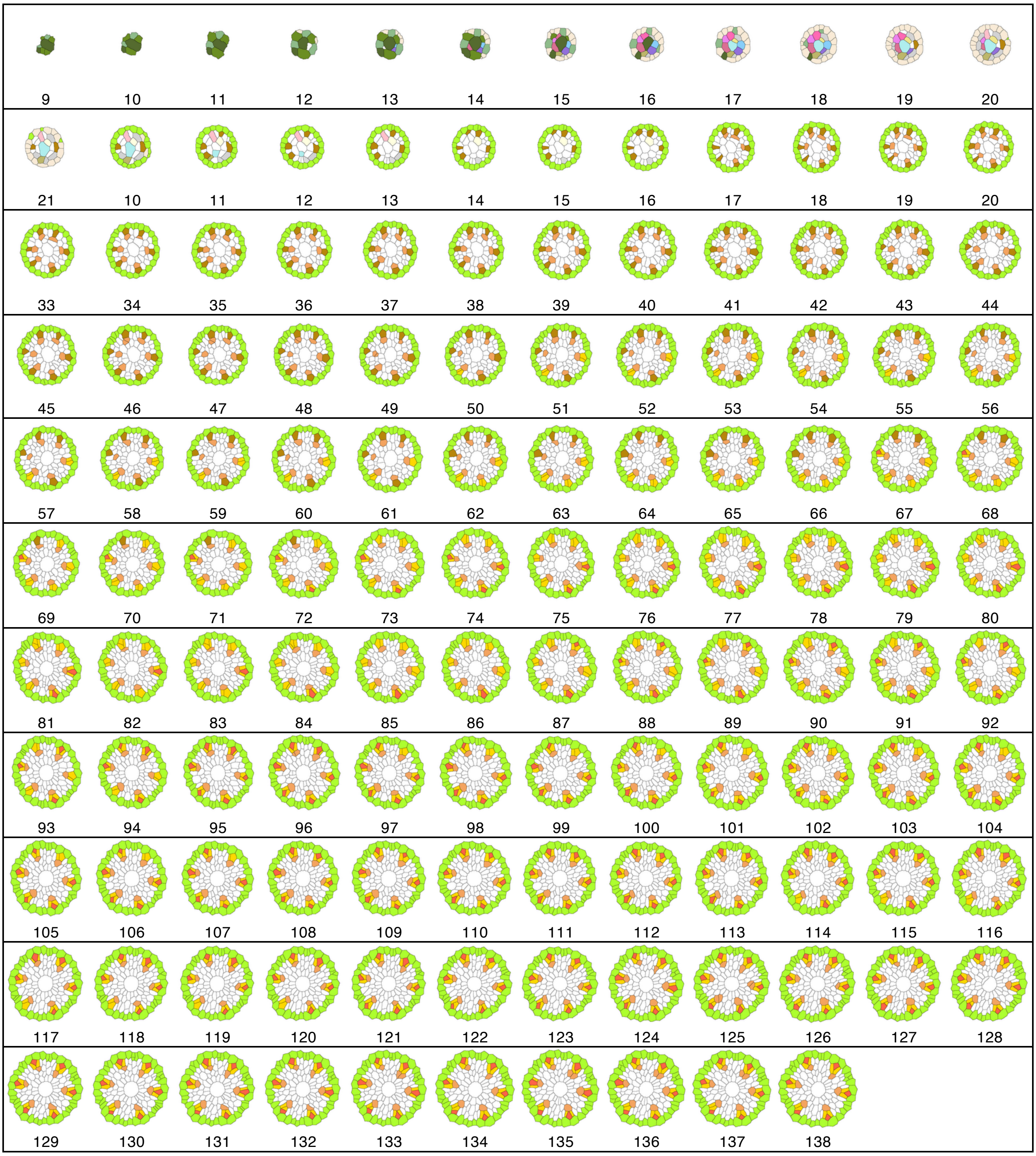

Supplementary Figure 1: Set of 1µm-thick serial sections colorized after contour extraction (Figure 3B) used to construct a 3D representation of the phloem system of a rice root.
