# Supplementary material for: Origin and Development of Phloem and Xylem as Revealed in 3D Models of the Vascular Cylinder in Primary Roots of Oryza sativa L. cv. Hitomebore
Source: Plants (Basel). 2026 Feb 14;15(4):607. doi: 10.3390/plants15040607 (PMC12944624; doi:10.3390/plants15040607)
Supplement: Supplementary file 1 [file plants-15-00607-s001.zip › SupplFigureS6.pdf]

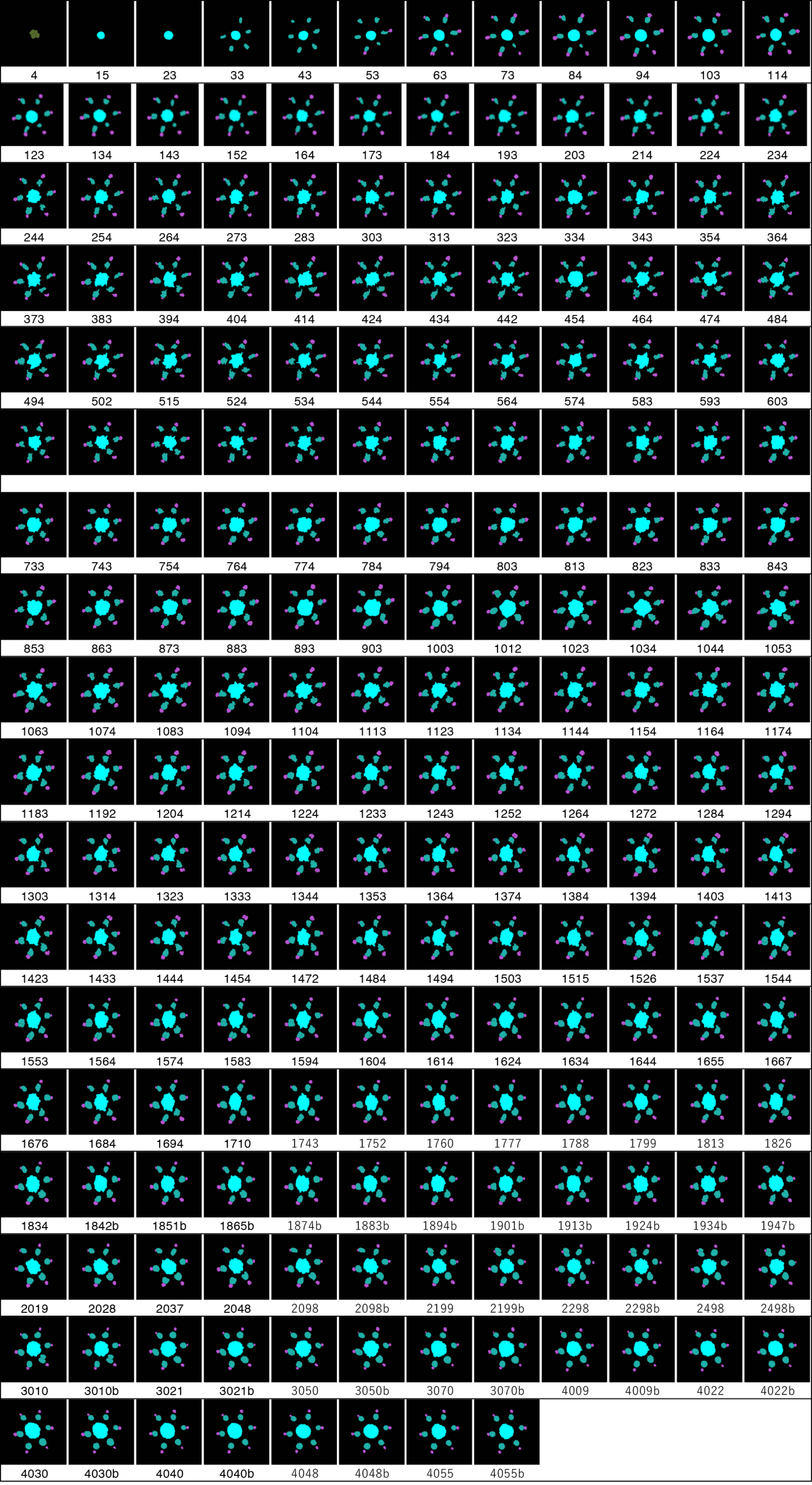

Supplementary Figure 6: Set of 2.5µm-thick colorized serial sections (Suppl. Fig, 5) after transparency was introduced to construct a 3D representation of xylem system of a rice root.
