# Supplementary figures and images for: Origin and Development of Phloem and Xylem as Revealed in 3D Models of the Vascular Cylinder in Primary Roots of Oryza sativa L. cv. Hitomebore
Source: Plants (Basel). 2026 Feb 14;15(4):607. doi: 10.3390/plants15040607 (PMC12944624; doi:10.3390/plants15040607)

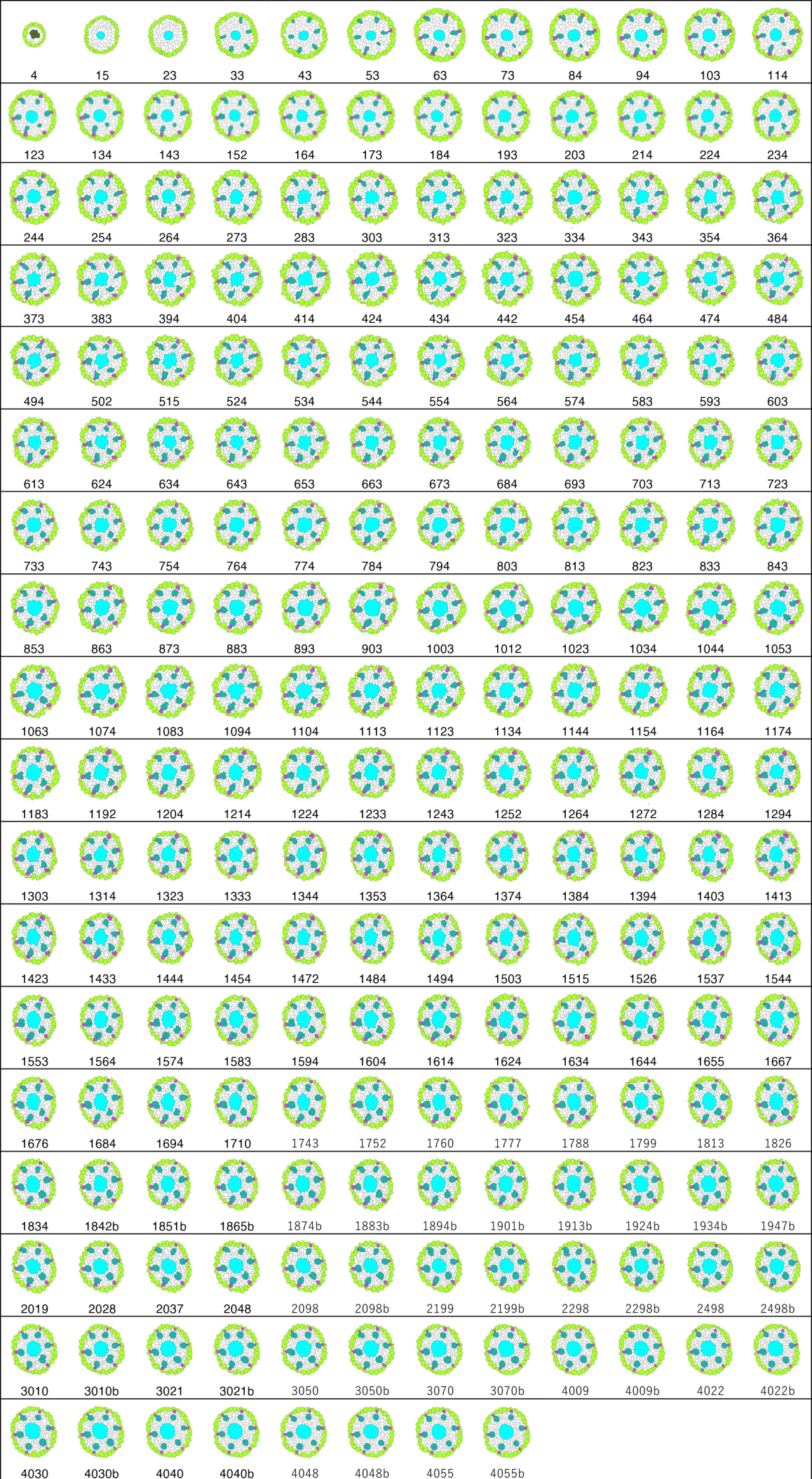

Supplement: Supplementary file 1 [file plants-15-00607-s001.zip › SupplFigureS5.pdf]
